# Filter Effects and Filter Artifacts in the Analysis of Electrophysiological Data

**DOI:** 10.3389/fpsyg.2012.00233

**Published:** 2012-07-09

**Authors:** Andreas Widmann, Erich Schröger

**Affiliations:** ^1^Institute of Psychology, University of LeipzigLeipzig, Germany

In a recent review, VanRullen (2011) concludes that electrophysiological data should not be filtered at all when one is interested in the temporal dynamics or onset latencies of the electrophysiological responses. This conclusion was based on the observation that response onset latency was “smeared out in time for several tens or even hundreds of milliseconds” (p. 6) in a simulated dataset.

It is correct that any band limitation in the frequency domain necessarily affects the signal in the time domain resulting in reduced precision and artifacts (cf. e.g., Luck, 2005). Nevertheless, here, we will discuss that the problem is overestimated by about an order of magnitude by the assumptions and analysis parameters used in VanRullen's simulated dataset and advertise the cautious usage of carefully designed filters to be able to also detect small signals.

## Filter Selection

**Figure A1:**
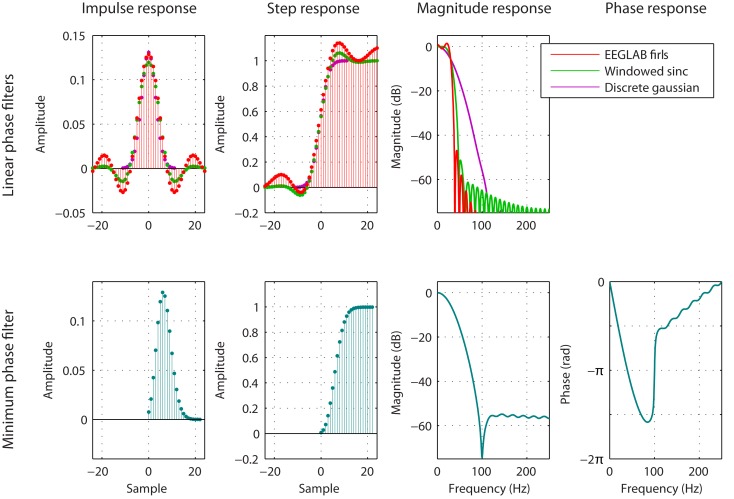
**Prototypical low-pass linear-phase filters’ impulse, step, magnitude, and phase responses (top row; sampling frequency = 500 Hz, cutoff frequency 30 Hz)**. The EEGLAB “Basic FIR filter” (red, 49 points, default settings, EEGLAB v11.0.2.1b; Delorme et al., 2011) exhibiting excessive ringing artifacts (“ripples” in the time domain observed if filtering a non-oscillating input, e.g., a step signal, yields an oscillating output) is shown in comparison to a windowed sinc (green, 49 points, firfilt EEGLAB plugin; Widmann, 2006) and a discrete Gaussian kernel filter (σ = 6.18 ms, based on a modified Bessel function; Lindeberg, 1990). The minimum-phase converted version of the discrete Gaussian kernel filter (bottom row; “causal” filter converted by means of Hilbert transform) shows a considerably non-linear phase response but does not show a response before signal onset.

The filter selected in VanRullen's simulation was a bad choice as it results in artifacts not related to filtering *per se*. The FIR filter generated by EEGLAB (Delorme et al., 2011) with default settings exhibits excessive filter ringing (cf., Figure A1 in Appendix), and excessive pass-band ripple including non-unity gain at DC (the step response never returns to one). These artifacts are due to a known misconception in FIR filter design in EEGLAB^1^. The artifacts are further amplified by filtering twice, forward and backward, to achieve zero-phase.

**Figure 1:**
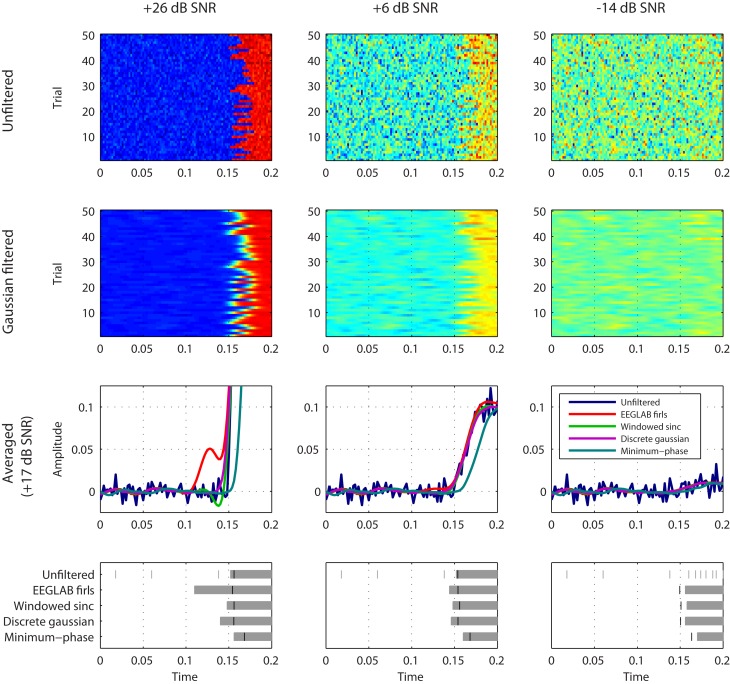
**Impact of filter type and signal-to-noise ratio (SNR) on the time course of the averaged signal and the detected signal onset latency in the simulated dataset (sampling frequency 500 Hz; step signal; signal onset 150–180 ms) as defined by VanRullen (2011)**. The simulated dataset was filtered with the EEGLAB firls based filter, a windowed sinc FIR filter (Widmann, 2006), a discrete Gaussian kernel filter (Lindeberg, 1990), and a minimum-phase converted version of the Gaussian filter (causal; see Figure A1 in Appendix for a detailed description of the filters). Single trial signal-to-noise ratio was reduced in 20 dB-steps from +26 dB (original dataset; left column) to −14 dB (right column). The Gaussian filtered single trials (second row) and the averaged trials (third row) are displayed. Signal onset latency was measured by a running one-sided t-test (bottom row; gray bars) and jack-knifing with a relative 20%-criterion (black lines; Kiesel et al., 2008).

With more appropriate filters the underestimation of signal onset latency due to the smoothing effect of low-pass filtering could be narrowed down to about 4–12 ms in the simulated dataset (see Figure 1 and Appendix for a simulation), that is, about an order of magnitude smaller than assumed.

## Signal-to Noise Ratio

The signal-to-noise ratio chosen by VanRullen for the simulated dataset is implausibly high (+26 dB at single trial level, +43 dB averaged) as signal-to-noise ratios smaller than one are common in real electrophysiological data. This assumption biases the conclusion on the detectability of the signal without filtering and overestimates the impact of filter ringing artifacts.

At more realistic signal-to-noise ratios no significant impact of the filter artifacts is observed (but only effects of transient smoothing by low-pass filtering; see Figure 1 and Appendix). The precision that can be achieved in the measurement of the response onset latency is limited by signal-to-noise ratio. Thus, the trade-off between filter effects versus the signal-to-noise ratio gain by filtering must be considered.

## Filter Effects vs. Filter Artifacts

We also recommend to distinguish between filter effects, that is, the obligatory effects any filter with equivalent properties – cutoff frequency, roll-off, ripple, and attenuation – would have on the data (e.g., smoothing of transients as demonstrated by the filter's step response), and filter artifacts, that is, effects which can be minimized by selection of filter type and parameters (e.g., ringing).

## Causal Filtering

In a commentary on VanRullen, Rousselet (2012) suggested to use “causal” filtering to solve the problem of signal onset latency underestimation due to smoothing. This is a valid recommendation, which has already been given (e.g., Luck, 2005). However, it should have been made explicit that the suggested type of “causal” filtering comes at the cost of a distortion of phase information also with FIR filters (cf., Figure A1 in Appendix).

The causality in filtering is not directly related to the symmetry of filter coefficients as implied in Figure 1 in Rousselet's (2012) comment. That is, the FIR filter labeled “non-causal” can also be applied in a causal way by not compensating the filter's delay (by not filtering the signal backward and not “left-shifting” the signal by the group delay). In order to reduce this filter delay in causal filtering, asymmetric “causal” FIR filters, more often referred to as minimum-phase filters, can be used. However, as FIR filter coefficients necessarily must be symmetric (or antisymmetric) for the filter to have linear-phase characteristic (Rabiner and Gold, 1975; Ifeachor and Jervis, 2002), this reduction of filter delay comes at the cost of a non-linear phase response and the introduction of a systematic delay in the signal (which can not easily be compensated due to non-linear phase). The recommendation for minimum-phase causal FIR filtering, thus, should be strictly limited to the detection of onset latencies and applications where causality is required for theoretical considerations. In its application it should be considered that the systematic delay and the non-linear phase response could also affect response onset information.

In the first paragraph of the appendix Rousselet (2012) suggests that the causal filtered signal could be left-shifted by the group delay to achieve zero-phase. We do not agree with this recommendation: First, this would re-introduce non-causality. Second, this statement is wrong as only linear-phase (anti-/symmetric FIR) filters can be made zero-phase by left-shifting the signal.

## Conclusion

In the analysis of electrophysiological data signal-to-noise ratio has to be improved by all adequate means. Priority should be given to the collection of higher numbers of trials and reduction of noise in data recording. However, in most situations filtering will nevertheless be necessary to appropriately analyze electrophysiological data. In these situations it is essential to know and understand the effects of filtering on the data and cautiously adjust filter settings (cutoff frequencies, roll-off, attenuation, and ripple) to the signal of interest and the particular application, e.g., by evaluating the effects of different filters on the data. Especially the high-pass filtering of slow ERP components or blinks, as commonly observed in the literature, might seriously affect ERP time course and amplitudes (see, Luck, 2005, for a detailed discussion). Furthermore, we recommend not using default filter settings, in particular when using EEGLAB, but rather to manually and carefully select filter type and parameters to minimize filter artifacts.

Filtering can result in considerable distortions of the time course (and amplitude) of a signal as demonstrated by VanRullen (2011). Thus, filtering should not be used lightly. However, if effects of filtering are cautiously considered and filter artifacts are minimized, a valid interpretation of the temporal dynamics of filtered electrophysiological data is possible and signals missed otherwise can be detected with filtering.

## Footnotes

^1^The EEGLAB “Basic FIR filter” function is based on the firls (least square fitting of FIR coefficients) MATLAB function (in the current version 11.0.2.1b as of writing this commentary). Filter length is defined independently of transition-band width. This can result in various adverse effects from sub-optimal stop-band attenuation, over filter artifacts, to leakage in the transition-band (the infamous “band-pass filter bug”). The problem is increased by the property of the firls function that transition-bands are defined as “do not care” regions. In a warning message it is announced in the current EEGLAB version that firls based filters are no longer recommended and fir1 should be used instead and will be the default setting in a future version. In its current implementation this change will not solve the problem as filter length and transition-band width are still defined independently. The filter actually generated by the fir1 function will deviate from the requested and reported transition-band width.

## Appendix

### Filter selection

We re-analyzed the simulated dataset as defined by VanRullen by means of a 49 point Hamming windowed sinc FIR filter (same length as the “default” EEGLAB generated filter; Widmann, 2006), and a discrete Gaussian kernel filter (σ = 6.18 ms; see Figure A1 for impulse, step, magnitude, and phase responses). The signal onset latency was underestimated by about 4 (windowed sinc) to 12 ms (discrete Gaussian) relative to unfiltered data compared to 42 ms when applying the EEGLAB firls default filter by one-sided *t*-tests (see Figure 1, bottom row; with non-simulated data more appropriate methods as, e.g., cluster-based non-parametric analysis, Maris and Oostenveld, 2007, could have been employed). No signal onset latency underestimation was observed using a jack-knifing technique with a relative 20%-criterion (Kiesel et al., 2008). Significant ringing artifacts could still be observed with the windowed sinc filter, in particular undershoot before signal onset (see Figure 1, left column, third row). Additionally, we re-analyzed the dataset by means of a causal filtering with a minimum-phase converted discrete Gaussian kernel filter as suggested by Rousselet (2012). The signal onset latency was overestimated by 4 ms due to the systematic delay introduced by causal filtering (16 ms as estimated by jack-knifing). However, the morphology of the signal was considerably affected by the non-linear phase response of the filter.

### Signal-to-noise ratio

In two additional analyses we reduced single trial signal-to-noise ratio in the simulated dataset in steps of −20 dB to +6 dB and −14 dB by reducing the signal amplitude from 1 to 0.1 and 0.01, respectively (Figure 1, columns two and three). At +6 dB signal-to-noise ratio the differences in onset latency underestimation between the linear-phase filters were significantly reduced (non-unity DC gain is still noticeable with the EEGLAB firls filter). No significant ringing artifacts were observed with the windowed sinc FIR filters. Importantly, at −14 dB single trial signal-to-noise ratio the signal could no longer be reliably detected without filtering and thus no signal onset latency could be determined. With non-causal and causal filtering the signal was detectable and the onset latency was overestimated by 4–6 and 18 ms, respectively (0 and 14 ms as estimated by jack-knifing). −14 dB single trial signal-to-noise ratio would be considered a good value in many electrophysiological measurements as, e.g., in electroencephalography (EEG). Averaging the 50 trials improved signal-to-noise ratio by +17 dB. Filtering further improved signal-to-noise ratio by about +12 dB allowing the reliable detection of the signal.

We would like to note that ringing artifacts must be considered in relation to noise level. In non-simulated electrophysiological data ringing artifacts are not expected to have a major impact due to the high noise level on the one hand and the absence of ultra-sharp transients on the other hand.

### Recommendations for selection of filter type and parameters

Unfortunately there cannot be given a ubiquitously valid recommendation for the selection of optimal filter settings, type, and parameters. They have to be individually adjusted to each application.

Infinite impulse response (IIR) filters are often considered as computationally more efficient compared to FIR filters as they are shorter. However, it should be considered, that the signal has to be filtered twice – forward and backward – to achieve zero-phase (possibly introducing artifacts with DC offsets at signal boundaries and squaring the frequency response); a larger number of computations is necessary with IIR filters due to recursive operation (relative to the IIR filter's shorter length); numerical errors can be accumulated due to the infinitive impulse response; and they are more difficult to control and can be unstable.

For FIR filters, only symmetric linear-phase filters should be considered for most applications in electrophysiology as they can be easily made zero-phase by left-shifting the signal by the filters group delay. There are various methods to design FIR filters, e.g., the Remez-exchange (equiripple) algorithm, preferable for arbitrary frequency responses not very common in the analysis of electrophysiological data (McClellan et al., 1973). The authors have good experiences with windowed sinc FIR filters, also commonly referred to as “ideal” filters due to the rectangular shape of the sinc function in the frequency domain. Implementations for the analysis of electrophysiological data can be found, e.g., in EEProbe software package (ANT, Enschede, The Netherlands) and the open-source firfilt EEGLAB plugin (Widmann, 2006). For a widely accessible introduction to windowed sinc FIR filter design see, e.g., Smith (1999). Windowed sinc FIR filter's stop-band attenuation (and pass-band ripple) can be precisely controlled by selection of window type; the filters’ transition-band width is a function of filter order/length (and window type), thus, filter length can be estimated (as with Remez-exchange FIR filters) or computed (with Kaiser windows), and high-pass filters can be easily optimized for excellent DC attenuation. If filter ringing is assumed to have an impact on a particular application, non-oscillating FIR filters, as, e.g., Gaussian kernel FIR filters, should be considered.

As rule of thumb, stop-band attenuation should be selected only as high as necessary, wider transition-bands (slow roll-off) should be preferred over narrower ones where possible. Cutoff frequencies and transition-bands should be separated from the signal of interest in the frequency domain to minimize distortion of the signal by filter artifacts and undesired filter effects. The filter should be as short as possible in order to minimize temporal smearing. Low-pass filters can sometimes be omitted in favor of later analysis steps introducing additional filtering as, e.g., computing time window mean values (representing low-pass filters as well). Balancing transition-band width and cutoff frequency is a particular challenge for high-pass filter design as the transition-band is limited by DC on the one hand but cutoff frequency should be low in order not to distort slow components on the other hand. Extreme cutoff frequencies <0.1 Hz as found sometimes in the literature should be avoided as filters usually become very long.
